# Temperature Tolerance and Stress Proteins as Mechanisms of Invasive Species Success

**DOI:** 10.1371/journal.pone.0014806

**Published:** 2011-04-26

**Authors:** Robyn A. Zerebecki, Cascade J. B. Sorte

**Affiliations:** 1 Marine Science Center, Northeastern University, Massachusetts, United States of America; 2 Bodega Marine Laboratory and Department of Evolution and Ecology, University of California at Davis, Bodega Bay, California, United States of America; California Academy of Sciences, United States of America

## Abstract

Invasive species are predicted to be more successful than natives as temperatures increase with climate change. However, few studies have examined the physiological mechanisms that theoretically underlie this differential success. Because correlative evidence suggests that invasiveness is related to the width of a species' latitudinal range, it has been assumed – but largely untested – that range width predicts breadth of habitat temperatures and physiological thermotolerances. In this study, we use empirical data from a marine community as a case study to address the hypotheses that (1) geographic temperature range attributes are related to temperature tolerance, leading to greater eurythermality in invasive species, and (2) stress protein expression is a subcellular mechanism that could contribute to differences in thermotolerance. We examined three native and six invasive species common in the subtidal epibenthic communities of California, USA. We assessed thermotolerance by exposing individuals to temperatures between 14°C and 31°C and determining the temperature lethal to 50% of individuals (LT_50_) after a 24 hour exposure. We found a strong positive relationship between the LT_50_ and both maximum habitat temperatures and the breadth of temperatures experience across the species' ranges. In addition, of the species in our study, invasives tended to inhabit broader habitat temperature ranges and higher maximum temperatures. Stress protein expression may contribute to these differences: the more thermotolerant, invasive species *Diplosoma listerianum* expressed higher levels of a 70-kDa heat-shock protein than the less thermotolerant, native *Distaplia occidentalis* for which levels declined sharply above the LT_50_. Our data highlight differences between native and invasive species with respect to organismal and cellular temperature tolerances. Future studies should address, across a broader phylogenetic and ecosystem scope, whether this physiological mechanism has facilitated the current success of invasive species and could lead to greater success of invasives than native species as global warming continues.

## Introduction

Two of the greatest threats to biodiversity and ecosystem functioning are species invasions and global climate change [1]–[3]. Both introduced species and climate change have, individually, had wide-ranging impacts on species' abundances, distributions, and interactions, and they have resulted in local extinctions [1], [4]–[11]. Furthermore, climate change and invasions may interact, with climate-change conditions favoring – and, thus, facilitating the spread of – non-native species [12]–[15]. The mechanism underlying this interaction between climate change and biological invasions, however, remains unclear. Here, we address temperature tolerance as a potential contributor to the success of invasive species, and we examine heat-shock protein (Hsp) expression as a physiological mechanism of this differential temperature tolerance.

**Figure 1:**
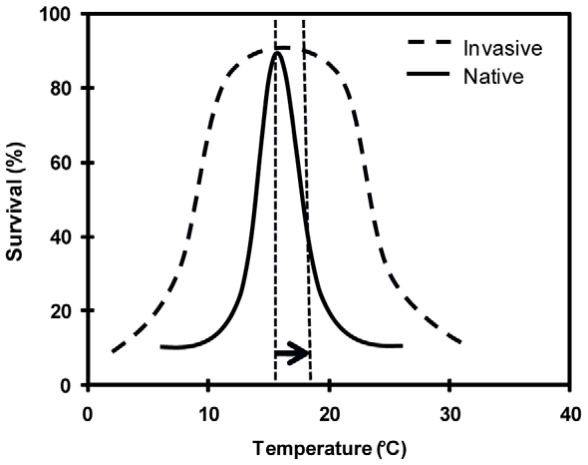
Conceptual model of greater invasive eurythermality. If an invasive (dashed line) has a broader range or higher limit of temperature tolerance than a native (solid line) then an increase in temperature (illustrated by a shift from the left to the right vertical dashed line) is likely to have a disproportionately negative impact on the native species. This physiological mechanism has the potential to precipitate changes in community composition, with increases and decreases in invasive and native relative abundance, respectively.

Eurythermality, or the ability to maintain physiological function over a wide range of temperatures, is a trait that is likely to be favored as the earth warms [5], [8] and may be enhanced in invasive, relative to native, species [10], [12], [16]–[18]. If invasive species have broader and/or greater physiological tolerances than natives occupying the same thermal habitat, it is likely that increased temperatures associated with climate change will exceed native species' tolerance limits before those of invasives (Figure 1). At the community level, this would lead to a compositional shift with a disproportionate increase in invasive, and decrease in native, species. To date, studies suggesting that invasives are more eurythermal than native species have typically relied on latitudinal range as a proxy for both range of habitat temperatures and physiological temperature tolerance [16], [19]–[22]. For example, Rejmánek [21] showed that, in two families of European herbaceous plants, species that have invaded North America have broader geographic ranges than those that have not invaded. However, the suggestion that these invasive species tolerate a wider range of climates and, therefore, may be favored by climate change has rarely been empirically tested [12],[20]. In the few studies that compared temperature tolerances between native and invasive species, results were either highly species-specific (e.g., [23]) or conflicting (e.g., [24]–[26]). In our study, we used geographic range data and global temperature databases to determine actual geographic temperature limits for each species, and we related habitat temperatures to empirically-determined temperature tolerances.

In addition to addressing eurythermality as a trait conferring success to invasive species, we examined the expression of heat-shock proteins (Hsps) as a potential subcellular mechanism underlying organismal temperature tolerances [27]–[29]. Heat-shock proteins (including the 70-kda Hsp that we studied, Hsp70) are molecular chaperones that assist in the refolding of denatured cellular proteins and, by so doing, minimize the cellular cost of protein degradation and replacement [27], [30]–[31]. Hsp70 expression can be both constitutive and stress-induced. The level of expression may, therefore, be related both to the innate ability of the organism to withstand environmental stresses and to the amount of stress-induced protein damage the organism has already experienced [31]–[34]. Recent studies have shown that cellular concentration of Hsp70 can be directly related to organismal temperature tolerance (e.g. [33]–[36]). Invasive species could have higher temperature tolerances if they express more Hsp70 constitutively or induce Hsp70 expression more quickly, and our study is one of the first to examine differences in Hsp expression between native and invasive species (see also [37]–[40]).

We addressed the hypothesis that invasives are more eurythermal than native species – and examined heat-shock protein expression as a potential underlying mechanism of thermotolerance – in the marine epibenthic community of Bodega Harbor, California, USA. This community is primarily composed of sessile organisms, including tunicates (sea squirts) and bryozoans, that colonize subtidal substrata such as docks, boat hulls, and natural rocky reefs. There has been a recent increase in biological invasions in epibenthic communities over the past several decades, both globally [41] and at our study site in Bodega Harbor, where local ocean temperature has also been increasing [15], [42]. We tested two specific hypotheses for nine common species that account for almost 80% of the occupied space in the Bodega Harbor epibenthic community. First, we examined whether geographic temperature limits are related to temperature tolerance, indicating greater eurythermality in invasive species, based on results from a lethal temperature tolerance experiment. Second, we used immunoblotting methods to quantify heat-shock protein expression in the native *Distaplia occidentalis* and invasive *Diplosoma listerianum* to assess whether heat-shock protein expression could be a subcellular mechanism underlying differences in temperature tolerance. Within this set of common species in a subtidal community, higher temperature tolerance in invasive species was related to geographic temperature range. Furthermore, our results for *Distaplia* and *Diplosoma* join a small, but growing, body of literature on the potential for Hsp70 expression to underlie temperature tolerance differences between native and invasive species.

## Results

**Figure 2:**
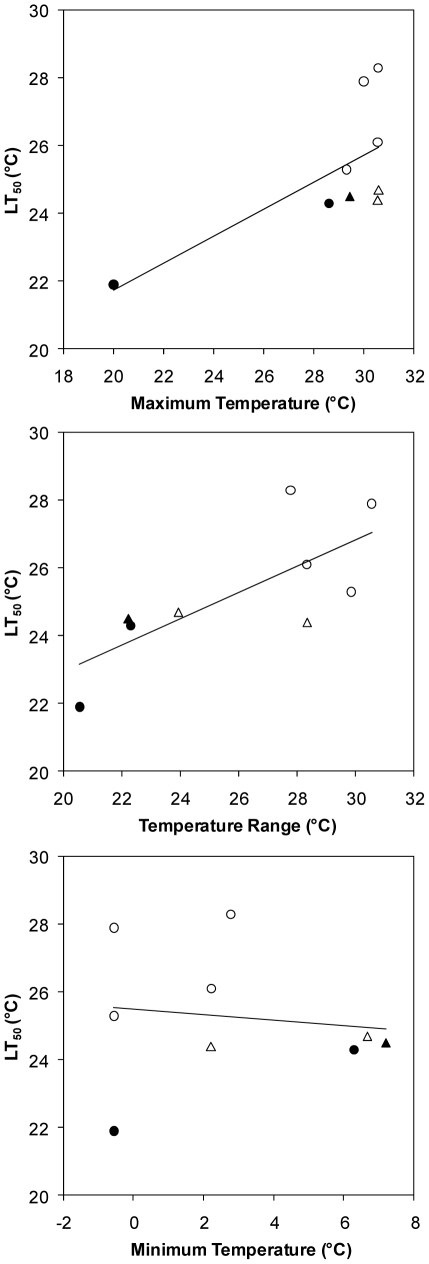
Relationship between lethal temperature tolerance and geographic temperature attributes. Each datapoint represents one of the 9 study species, including 3 natives (closed symbols) and 6 invasives (open symbols) within 2 phyla: tunicates (circles) and bryozoans (triangles). Higher LT_50_ values were associated with higher maximum temperatures (linear regression, *p* = 0.039) and broader geographic temperature ranges (*p* = 0.022), calculated by subtracting the minimum monthly mean temperature from the maximum monthly mean temperature across the species' ranges (see Table 1 and Materials and Methods), but were not related to minimum temperatures.

In individuals from this California subtidal epibenthic community, higher temperature tolerances were related to broader geographic temperature ranges (linear regression, *F*_1,7_ = 8.54, *p* = 0.022) and higher maximum habitat temperatures (*F*_1,7_ = 6.44, *p* = 0.039) across these species' ranges (Figure 2). Minimum habitat temperature was unrelated to this measure of a species' upper lethal temperature (*p* = 0.738). Habitat temperature breadth was slightly more strongly related to maximum (*p* = 0.087) than minimum (*p* = 0.139) habitat temperature.

**Figure 3:**
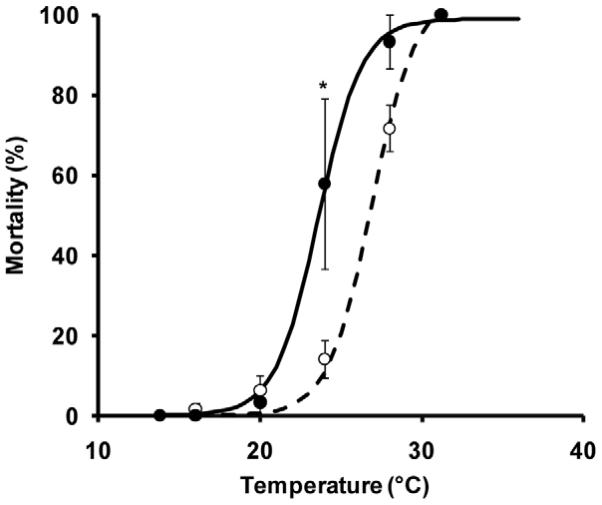
Native and invasive species temperature tolerance. Temperature tolerance of native (closed symbols, solid line; *n* = 3) and invasive species (open symbols, dashed line; *n* = 6) shown as mean (±1 SE) mortality after a 24 h exposure to six temperature treatments between 14 and 31°C. Logistic curves indicate the higher average LT_50_ for invasives (dashed line) than natives (solid line) due to the difference in mortality at 24°C (*, *p*<0.05).

The greater average LT_50_ of invasives compared with native species in our study was driven by differences at 24°C, where mortality was only 14.0% (±6.11 SE) for invasives but was 57.8% (±21.20) for natives (t-test, *t* = 2.649, *p* = 0.033; Figure 3). Mortality curves for individual species are available in Figure S1 in the supporting information. Overall, the invasive species inhabited locations with, on average, broader temperature ranges (28.1°C ±0.9) than the natives (21.7°C ±0.6; *p* = 0.003). The invasives also tended to live at higher maximum temperatures (30.3°C ±0.2 for invasives; 26.0°C ±3.0 for natives; *p* = 0.069).

**Figure 4:**
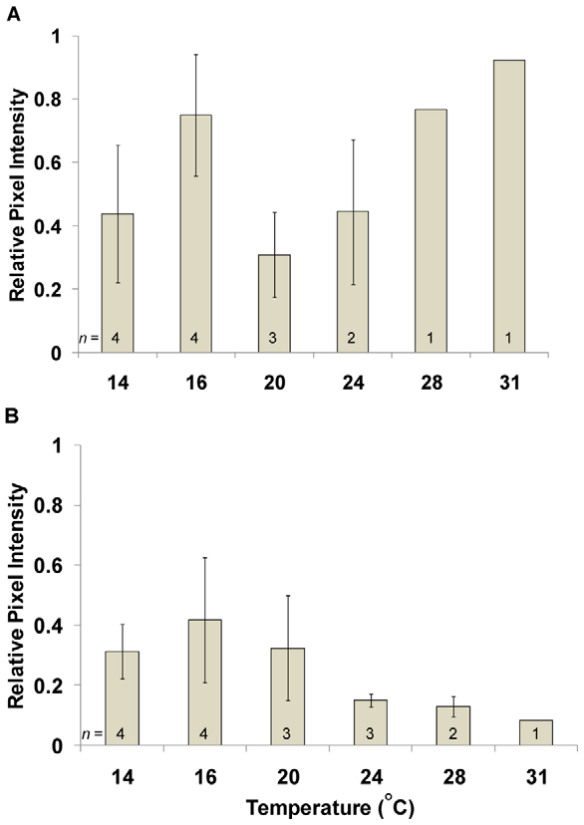
Level of heat-shock protein (Hsp70) in the invasive *Diplosoma* (A) and native *Distaplia* (B). Total Hsp70 (shown as mean [±1 SE] immunoblot pixel intensity relative to an internal standard) in *Diplosoma* (A) and *Distaplia* (B) individuals after a 24 h exposure to 6 treatment temperatures and a 48 h recovery period at ambient temperature. Sample sizes are shown at the base of the columns.

Our analysis of expression of a 70-kda heat-shock protein (Hsp70), a potential subcellular mechanism of organismal temperature tolerance, indicated that Hsp70 levels were higher in the more thermotolerant, invasive tunicate *Diplosoma* than in the less thermotolerant, native *Distaplia* (ANOVA, *F*_1,20_ = 8.065, *p* = 0.010; Figure 4). For *Distaplia*, Hsp70 levels were about twice as high in individuals exposed to temperatures below the species' LT_50_ of 21.9°C than above this threshold (t-test, *t* = 1.855, *p* = 0.083; Figure 4). For *Diplosoma*, we were limited by a low sample size above its LT_50_ of 27.9°C, which approached our maximum treatment temperature; however, a decline in Hsp70 expression was not observed at increased temperature for this species.

## Discussion

We provide empirical data which indicate that, for species in a subtidal epibenthic community, attributes of geographic temperature ranges are related to higher temperature tolerances. Among these species, invasives tend to inhabit locations with broader temperature ranges and higher maximum temperatures than native species. Range width has been suggested as a general characteristic of invasion success [16], [19], [21], attributable either to greater physiological tolerance (e.g., eurythermality), increased propagule pressure (see [43]), or both. Because introduced species have to pass through several abiotic (and biotic) filters in the multi-stage invasion process, species with broader physiological tolerances may be more likely to survive and become established as invaders [44]–[46]. At the same time, species that are able to occupy larger areas may have more source populations, increasing their likelihood of being transported elsewhere [43], [46]. Our results lend some support to this physiological tolerance hypothesis, without discounting a role for increased propagule pressure, and have implications for the relative impacts of climate change on invasive and native species. To that end, given that (1) broad ranges are characteristic of invaders [20], and (2) we found that temperature tolerance increases with geographic temperature range (across both native and invaded ranges), the possibility that invasive species could be poised for greater success with climate change merits close consideration.

Indeed, a direct comparison of these species in a temperate epibenthic community revealed that invasives are more tolerant of higher temperatures than natives on average (also see [15]). The temperature *versus* tolerance relationships and differences between natives and invasives were driven by the tunicate species data. For example, as far as tolerance rank, the least tolerant species were natives (Table 1), with the exception of the native bryozoan *Bugula californica*. We suspect that an age effect may partly explain why *B. californica* – which had to be hand collected at a more advanced stage due to low recruitment – appeared to be the most thermotolerant native species, when the other two bryozoans (*Bugula neritina* and *Watersipora subtorquata*) were the least thermotolerant invasives. A positive relationship between age and temperature tolerance (e.g., [47], [48]), and between age and survival [49], has been shown for many taxonomic groups, and most *B. californica* individuals in the LT_50_ experiment were ∼3–4 weeks older than individuals of the other 8 species. A possible influence of age on *B. californica* temperature tolerance was indicated by an *ad hoc* comparison of the tolerance of younger (natural recruitment) and older (dock-collected) individuals. At 20, 24, and 28°C, mortality in replicates composed of younger individuals (followed by sample size) was 50% (1), 50% (2), and 100% (2), respectively, whereas mortality of older individuals –0% (4), 33% (3), and 67% (3) – was lower at all three treatment temperatures. Although this trend was not significant at any temperature (t-test, *p*>0.2), if age effects led to an increase in LT_50_ for *B. californica*, then we would have underestimated the difference in LT_50_ values between native and invasive species. Alternatively, such departures from the general pattern of higher tolerance in invasives highlight the importance of broadening the phylogenetic scope of physiological tolerance studies of invasion success.

**Table 1:** Geographic temperature limits and lethal temperatures (LT_50_) for the 9 study species.

			Limits of Geographic Temperature Range		Temp. Limits (°C)		
Species	Origin	Taxon	Minimum	Maximum	Min.	Max.	LT_50_
*Distaplia occidentalis*	Native	Tunicate	Anchorage, AK, USA	La Jolla, CA, USA [^66^]	-0.6	20.0	21.9
*Ascidia ceratodes*	Native	Tunicate	Departure Bay, BC, Canada	Panama City, Panama 1	6.3	28.6	24.3
*Bugula neritina*	Invasive	Bryozoan	Boston, MA, USA [^69^]	Key West, FL, USA	2.2	30.6	24.4
*Bugula californica*	Native	Bryozoan	Cape St. James, BC, Canada	Grand Isle, LA, USA	7.2	29.4	24.5
*Watersipora subtorquata*	Invasive	Bryozoan	Port Townsend, WA, USA [^72^]	Red Sea [^67^] ^,^ 2	6.7	30.6	24.7
*Botrylloides violaceus*	Invasive	Tunicate	Anchorage, AK, USA [^67^]	Palau 2	-0.6	29.3	25.3
*Didemnum vexillum*	Invasive	Tunicate	Bar Harbor, ME, USA [^67^]	Key West, FL, USA	2.2	30.6	26.1
*Diplosoma listerianum*	Invasive	Tunicate	Anchorage, AK, USA	Miami Beach, FL, USA	-0.6	30.0	27.9
*Botryllus schlosseri*	Invasive	Tunicate	Eastport, ME, USA	Naples, FL, USA	2.8	30.6	28.3

1 M. Carman, *pers. comm.*; temperature data were from the Smithsonian Tropical Research Institute [76].

2 Temperature data were from the World Ocean Atlas [73].

Species are in order of increasing LT_50_ (temperature lethal to 50% of individuals). Geographic limits are from the Ocean Biogeographic Information System [71], and temperature measurements are from NOAA tide gauge [74] and Canadian lighthouse data [75], except where indicated by footnotes. Temperature limits are the minimum and maximum monthly means at these geographic temperature limits (see Materials and Methods).

To assess the role of tolerance in invasion success and whether invasive species may be favored in a changing climate, it is also important to consider our temperature tolerance results concurrent with studies of additional physiological and demographic processes. For example, these results corroborate conclusions from the same system based on differential temperature effects on recruitment [14], [50], as well as on growth and survival [15]. Recruitment is positively related to temperature for 5 of the 6 invasives but not for the 2 natives (all but *Ascidia*) considered here [50]. In addition, when mean temperature was increased by 4.5°C, survival was not affected for invasives but was decreased for the one native considered (*Distaplia*), and temperature impacts on growth rates were, in general, more positive for invasives than natives [15].

In Bodega Harbor, species with the lowest temperature tolerances may be the most susceptible to global warming, whereas abundances of species with the highest temperature tolerances may be most augmented. The common native *Distaplia* is likely to experience the greatest reduction in abundance as not only does it have the lowest LT_50_ (Table 1) but, in relation to invasives, it also experienced higher mortality and lower enhancement of growth when mean temperature was increased by 4.5°C [15]. Conversely, the invasive tunicates *Diplosoma* and *Botryllus schlosseri* had the highest LT_50_ values, and they experienced decreases in survival and increases in growth rate at +4.5°C ([15]; M. Cockrell & C. Sorte *unpubl. data*). Among this set of species, there was no indication of an inverse relationship between thermotolerance and habitat temperature, as has been documented in several other systems [51]–[54], either across their geographic ranges or in Bodega Harbor (where they all inhabit the same maximum temperatures). However, a study of geographic variation in temperature tolerance of four of these invasive species (*Botrylloides, Botryllus, Diplosoma,* and *B. neritina*) did find evidence of more tolerant species living closer to their tolerance limits, suggesting the need for comparative studies to detect such a relationship at a geographic scale [55].

The expression of heat-shock proteins is a subcellular mechanism that could be partly responsible for differences in organismal responses to increased temperature. In our inter- and intra-specific comparisons of expression by the tunicates *Distaplia* and *Diplosoma*, we found that Hsp70 levels were higher in the invasive *Diplosoma* than in the native *Distaplia*, corresponding with the invasive's relatively greater temperature tolerance. Because individuals of these two species had the same thermal history, this variation between species is not likely due to differential heat-hardening but, rather, may represent either higher constitutive, or more rapidly induced, Hsp expression in *Diplosoma.* In *Distaplia*, individuals exposed to temperatures below the species' LT_50_ had relatively high Hsp70 levels and low mortality rates; however, in individuals exposed to temperatures above the LT_50_, Hsp70 levels were decreased approximately by half and mortality was high. These results are consistent with previous studies that have illustrated a positive relationship between Hsp70 expression and temperature tolerance (see [27], [56]). This is among the first sets of studies to address the role of the heat-shock response in explaining differences in physiological tolerances between native and invasive species. Hofmann & Somero [37] and Braby [38] both demonstrated greater induction of Hsp70, as well as a higher Hsp70 induction temperature, in the invasive mussel *Mytilus galloprovincialis* than its native congener *Mytilus trossulus.* In addition, Henkel et al. [40] found a greater increase in *hsp70* gene expression in the invasive kelp *Undaria pinnatifida* than in two native California species. Studies have also revealed alternative mechanisms of native *versus* invasive physiological tolerances, including enzyme function [57] and heart function [58]. Together, these comparisons suggest that invasives may currently have a greater capacity to deal with thermal stress than native species.

As ocean temperatures continue to increase in Bodega Harbor, as well as worldwide, it is possible that the resident epibenthic species could evolve increased temperature tolerances. However, no study to date has examined the possibility of adaptation to climate change in tunicate or bryozoan species, even though local adaptation, including of temperature tolerance [59], has been observed over distances of <60 km owing to relatively short dispersal distances of many epibenthic species [59], [60]. Local adaptation could help to explain the finding that for 8 out of 9 species, LT_50_ values for California populations were lower than the maximum habitat temperatures across the species' ranges. In fact, populations of *B. neritina, Botrylloides, Botryllus,* and *Diplosoma* living on the USA east coast had higher tolerance limits than these populations in California [55].

Adaptive potential should be increased in species with shorter generation times and with greater phenotypic – suggesting greater genetic – variation. Generation times are short in these species; for example, *Botryllus* can reach sexual maturity in 1 month [61], and populations in Maine, USA sexually reproduce 1–2 times per year [60]. Intraspecific phenotypic variation in temperature tolerance is apparent as differential mortality among individuals in the species mortality plots (Figure S1). All individuals of the 9 species experienced 100% mortality at 31°C. However, some survival in response to a 28°C exposure was observed for all 6 invasives and for 1 of 3 natives (*B. californica*), and some individuals of all invasive species and 2 of 3 natives survived at 24°C (Figure S1). Conversely, *Distaplia* showed no individual variation in mortality across any of the temperature treatments (average mortality at each temperature was either 0% or 100%), suggesting limited variation and, consequently, limited adaptive potential. Thus, even if adaptation occurs in these epibenthic species, the 6 invasives seem to have both an initial advantage over the natives, due to their current higher LT_50_ values, and a potential adaptive advantage because of their greater range of responses to the temperature exposures.

In conclusion, we present data from nine of the most common species in a subtidal community that serve as a starting point for examining the hypothesis that habitat temperatures across the species' ranges are associated with temperature tolerance. Our comparison of native and invasive tunicate species indicates the possibility that differences in Hsp70 expression could partly underlie the higher temperature tolerance of invasives. Future studies that include a greater number of species will help to clarify whether greater temperature tolerance and higher Hsp70 expression are general characteristics of invasive species *per se*.

Temperature tolerance differences between natives and invasives may be especially important in determining the impacts of extreme high temperature events which are predicted to increase in frequency and severity over the next decade [9], [62]. Although daily average temperatures in Bodega Harbor typically ranged from 9–17°C (between the years 2005–9; [50]), well below the LT_50_ of all 9 epibenthic species, a heat wave in July 2006 led to peak hourly temperatures of almost 20°C, with 19°C sustained over a 24 h period [63]. If, as predicted by climate models, mean ocean temperature increases by 3–4°C [9], [64], and heat wave severity increases by ∼2°C [62], over the next century, then Bodega Harbor temperatures during extreme events could exceed the LT_50_ of *Distaplia* (21.9°C) within the near future and of all three natives (Table 1) by the end of the 21^st^ century. Further studies that address the extent to which these findings for common species in a marine epibenthic community reflect a general pattern will continue to elucidate whether differences in eurythermality, temperature tolerance, and Hsp expression may predispose invasive species for greater success as global temperatures increase.

## Materials and Methods

We focused our study on nine species from the epibenthic community of Bodega Harbor, Bodega Bay, California (38.3290° N, 123.0581° W), including three natives and six invasive species from two phyla (Table 1). These 9 species accounted for approximately 79% of occupied space (excluding mussels which act, themselves, as substrate) based on a survey we conducted of the adult dock community.

### Geographic range and temperature limits

We determined the geographic range of each focus species by consulting primary literature, field guides and an online database (e.g. [65]–[72], M. Carman *pers. comm.*). Within each species' geographic range, we identified the geographic temperature range using data provided by the NOAA National Oceanographic Data Center [73], [74], Canadian Department of Fisheries and Oceans [75], and Smithsonian Tropical Research Institute [76]. Minimum temperatures were average monthly temperatures from the coldest months, and maximum temperatures were from the warmest months, across the species' ranges; neither were necessarily experienced at the latitudinal extremes. We used t-tests to compare the geographic temperature ranges of native and invasive species, and we used linear regression to relate temperature range and limits to temperature tolerance (LT_50_).

### Lethal temperature experiment

Individuals (≤5 weeks old) of our 9 focus species settled naturally on plastic tiles (Duplos, The LEGO© Group, Billund, Denmark) suspended 1 m below the dock surface at Spud Point Marina, Bodega Harbor. The experimental individuals, thus, started the experiment with the same thermal history. Tiles were transferred to the lab, counted, and weeded to contain a maximum of two individuals (subsamples) of a single focus species. Tiles with only one individual were used when necessary. The exception was that for some replicates of *B. californica*, due to low levels of natural recruitment, colonies were collected from the docks by hand and were connected to tiles with wire.

Individuals were acclimated in the running seawater table at approximately 12°C for 24 h prior to the experiment. During the experiment, tiles were suspended in separate containers (1 L). Temperature was raised gradually (1°C per 15 min) to 6 treatment temperatures (approx. 14, 16, 20, 24, 28, and 31°C) using aquarium heaters (7.5W, #T10401, Hydor USA, Sacramento, CA, USA). Each species × temperature combination was replicated 5 times with the exceptions that for *B. neritina*, *n* = 7 at 20 and 24°C, and *Ascidia* were only exposed to 20, 24, and 28°C (n = 3, 3 and 2, respectively) due to low recruitment. Species × temperature combinations were randomly assigned to tanks and experimental runs (9 runs over a 4 wk period). Water temperatures were monitored using a handheld digital thermometer (HH21, Omega Engineering, Inc., Stamford, CT, USA), and the average treatment temperatures across all replicates were calculated and used in the LT_50_ analyses.

Survival was determined by zooid responsiveness, with individuals scored as ‘live’ or ‘dead’. Tiles were examined immediately after the 24 hr temperature exposure and after 2 days of recovery in ambient running seawater. Because survival values did not vary with recovery time, we used the first observation for all analyses. We calculated species' LT_50_ values using Probit analysis, and t-tests were used to compare survival of native and invasive species at each temperature treatment. All data were analyzed using SAS v.9.1 (SAS Institute Inc., Cary, NC, USA).

### Heat-shock protein analysis

Hsp70 expression patterns across treatment temperatures were quantified for two species of colonial tunicates: the native *Distaplia* and invasive *Diplosoma*. Individuals used in the LT_50_ experiment were flash frozen in liquid nitrogen after 2 days of recovery in running seawater, and tissue was stored at −80°C. We successfully detected Hsp70 in 3 species (*Distaplia*, *Diplosoma,* and *Didemnum*) but not in 3 other species (*Botrylloides*, *Botryllus,* and the cryptogenic hydroid *Obelia* sp.) with the primary antibodies tested (SPA-822 and SPA-805, StressGen, Victoria, BC, Canada).

Samples were prepared by vortexing with glass beads in 30–50 µL of homogenization buffer. The homogenate was heated in a water bath for 5 min at 100°C and centrifuged at 14000 rpm for 15 min. Supernatant protein levels were determined using a BCA protein assay with amounts modified for a NANO-Drop 1000 spectrophotometer (both by Thermo Fisher Scientific Inc., Wilmington, DE, USA). Samples (20 µg protein) were run on a 10% polyacrylamide gel and transferred to nitrocellulose membrane (75 min at 80V). Membranes were stored overnight in blocking solution (0.1% Tween−20+5% Non-fat dry milk in TBS [Tris buffer]).

Hsp70 was detected by western blotting. The primary antibody was a mouse monoclonal anti-HSP70 (1∶1000, 90 min; SPA-822, StressGen, Victoria, BC, Canada) and secondary antibody was a goat anti-mouse IgG (1∶1000, 60 min; SAB-100, StressGen, Victoria, BC, Canada). Hsp70 was visualized using Enhanced Chemiluminescent reagents (ECL-Plus, GE Healthcare, Piscataway, NJ, USA) and quantified using Adobe Photoshop CS3 v.10.0.1 (Adobe Systems Inc., San Jose, CA, USA). Hsp70 levels reported are relative to a positive control (field-collected *Didemnum*), and relative pixel intensity was compared between species and temperature treatments with a 2-way ANOVA (including species, temperature, and their interaction) run in SAS. All data are presented as means ±1 SE.

## Supporting Information

Figure S1 Mean (±1 SE) mortality at 6 treatment temperatures between 14 and 31 degrees Celsius for each species (A, B. neritina; B, Watersipora; C, Botrylloides; D, Didemnum; E, Diplosoma; F, Botryllus; G, Distaplia; H, Ascidia; I, B. californica). LT50 after a 24 h exposure is denoted by the dashed lines.
(0.38 MB TIF)

## References

[1] Vitousek PM, Mooney HA, Lubchenco J, Mellilo JM (1997). Human domination of Earth's ecosystems.. Science.

[2] Sala OE, Chapin FS, Armesto JJ, Berlow E, Bloomfield J (2000). Global biodiversity scenarios for the year 2100.. Science.

[3] Halpern BS, Walbridge S, Selkoe KA, Kappel CV, Micheli F (2008). A global map of human impact on marine ecosystems.. Science.

[4] Ruiz GM, Fofonoff P, Hines AH, Grosholz ED (1999). Non-indigenous species as stressors in estuarine and marine communities: Assessing invasion impacts and interactions.. Limnol Oceanogr.

[5] Carlton JT, Mooney JT, Hobbs RJ (2000). Global change and biological invasions.. Invasive species in a changing world.

[6] Grosholz E (2002). Ecological and evolutionary consequences of coastal invasions.. Trends Ecol Evol.

[7] Walther GR, Post E, Convey P, Menzel A, Parmesan C (2002). Ecological response to recent climate change.. Nature.

[8] Parmesan C (2006). Ecological and evolutionary responses to recent climate change.. Annu Rev Ecol Syst.

[9] Intergovernmental Panel on Climate Change (2007). Climate Change 2007: The Physical Science Basis..

[10] Hellman JJ, Byers JE, Bierwagen BG, Dukes JS (2008). Five potential consequences of climate change for invasive species.. Conserv Biol.

[11] Dijkstra JA, Harris LG (2009). Maintenance of diversity altered by a shift in dominant species: implications for species coexistence.. Mar Ecol Progr Ser.

[12] Dukes JS, Mooney HA (1999). Does global change increase the success of biological invaders?. Trends Ecol Evol.

[13] Byers JE (2002). Impact of non-indigenous species on natives enhanced by anthropogenic alteration of selection regimes.. OIKOS.

[14] Stachowicz JJ, Terwin JR, Whitlach RB, Osman RW (2002). Linking climate change and biological invasions: ocean warming facilitates nonindigenous species invasions.. Proc Natl Acad Sci USA.

[15] Sorte CJB, Williams SL, Zerebecki RA (2010). Ocean warming increases threat of invasive species in a marine fouling community.. Ecology.

[16] Rejmánek M (2000). Invasive plants: approaches and predictions.. Aust Ecol.

[17] Kolar CS, Lodge DM (2001). Progress in invasion biology: predicting invaders.. Trends Ecol Evol.

[18] Rahel FJ, Bierwagen B, Taniguchi Y (2008). Managing aquatic species for conservation concern in the face of climate change and invasive species.. Conserv Biol.

[19] Forcella F, Wood JT (1984). Colonization potential of alien weeds are relative to their “native” distributions: implications for plant quarantine.. J Aust Inst Agric Sci.

[20] Rejmánek M, Pyšek P, Prach K, Rejmánek M, Wade M (1995). What makes a species invasive?. Plant invasions: general aspects and special problems.

[21] Rejmánek M (1996). A theory of seed plant invasiveness: The first sketch.. Biol Conserv.

[22] Rejmánek M, Richardson DM (1996). What attributes make some plant species more invasive?. Ecology.

[23] Carveth CJ, Widmer AM, Bonar SA (2006). Comparison of upper thermal tolerances of native and nonnative fish species in Arizona.. Trans Amer Fish Soc.

[24] Human KG, Gordon DM (1996). Exploitation and interference competition between invasive Argentine ant, *Linepithema humile*, and native ant species.. Oecologia.

[25] McMahon RF (2002). Evolutionary and physiological adaptations of aquatic invasive animals: r selection versus resistance.. Can J Fish Aquat Sci.

[26] Holway DA, Suarez AV, Case TJ (2002). Role of abiotic factors in governing susceptibility to invasion: a test with Argentine ants.. Ecology.

[27] Feder ME, Hofmann GE (1999). Heat-shock proteins, molecular chaperones, and the stress response.. Annu Rev Physiol.

[28] Hochachka PW, Somero GN (2002). Biochemical adaptation: mechanism and process in physiological evolution..

[29] Tomanek L (2008). The importance of physiological limits in determining biogeographical range shifts due to global climate change: the heat shock response.. Physiol Biochem Zool.

[30] Lindquist S (1986). The heat-shock response.. Annu Rev Biochem.

[31] Parsell DA, Lindquist S (1993). The function of heat-shock proteins in stress tolerance: degradation and reactivation of damaged proteins.. Annu Rev Genet.

[32] Roberts DA, Hofmann GE, Somero GN (1997). Heat-shock protein expression in *Mytilus californianus*: acclimatization (seasonal and tidal-height comparisons) and acclimation effects.. Biol Bull.

[33] Sørensen JG, Kristensen TN, Loeschcke V (2003). The evolutionary and ecological role of heat shock proteins.. Ecol Lett.

[34] Sorte CJB, Hofmann GE (2005). Thermotolerance and heat-shock protein expression in Northeastern Pacific *Nucella* species with different biogeographical ranges.. Mar Biol.

[35] Sanders BM, Hope C, Pascoe VM, Martin LS (1991). Characterization of the stress protein response in two species of *Collisella* limpets with different temperature tolerances.. Physiol Zool.

[36] Sun W, Montagu MV, Verbruggen N (2002). Small heat shock proteins and stress tolerance in plants.. Biochim Biophys Acta Gene Struct Expr.

[37] Hofmann GE, Somero GN (1996). Interspecific variation in thermal denaturation of proteins in the congeneric mussels *Mytilus trossulus* and *M. galloprovincialis*: evidence from the heat-shock response and protein ubiquitination.. Mar Biol.

[38] Braby CE (2004). Physiological ecology of native and invasive blue mussels (genus *Mytilus*) in central California..

[39] Henkel SK, Hofmann GE (2008). Differing patterns of *hsp70* gene expression in invasive and native kelp species: evidence for acclimation-induced variation.. J Appl Phycol.

[40] Henkel SK, Kawai H, Hofmann GE (2009). Interspecific and interhabitat variation in *hsp70* gene expression in native and invasive kelp populations.. Mar Ecol Progr Ser.

[41] Lambert G (2007). Invasive sea squirts: A growing global problem.. J Exp Mar Biol Ecol.

[42] Boyd MJ (1972). Fouling community structure and development in Bodega Harbor, California..

[43] Pyšek P, Jarošik V, Pergl J, Randall R, Chytry M (2009). The global invasion success of Central European plants is related to distribution characteristics in their native range and species traits.. Diversity Distrib.

[44] Mack RN, Simberloff D, Lonsdale WM, Evans H, Clout M (2000). Biotic invasions: causes, epidemiology, global consequences and control.. Ecol Appl.

[45] Theoharides KA, Dukes JS (2007). Plant invasion across space and time: factors affecting nonindigenous species success during four stages of invasion.. New Phyt.

[46] Olyarnik SV, Bracken MES, Byrnes JE, Hughes AR, Hultgren KM, Rilov G, Crooks JA (2009). Ecological factors affecting community invasibility.. Biological invasions in marine ecosystems: ecological, management, and geographic perspectives.

[47] Winne CT, Keck MB (2005). Intraspecific differences in thermal tolerance of the diamondback watersnake (*Nerodia rhombifer*): effects on ontogeny, latitude and sex.. Comp Biochem Physiol Mol Integr Physiol.

[48] Bowler K, Terblanche JS (2008). Insect thermal tolerance: what is the role of ontogeny, ageing and senescence?. Biol Rev.

[49] Gosselin L, Qian P (1997). Juvenile mortality in benthic marine invertebrates.. Marine Ecol Progr Ser.

[50] Sorte CJB, Stachowicz JJ (*in review*) Patterns and processes of compositional change in a California epibenthic community..

[51] Stillman JH, Somero GN (2000). A comparative analysis of the upper thermal tolerance limits of eastern Pacific porcelain crabs, genus *Petrolisthes*: influences of latitude, vertical zonation, acclimation, and phylogeny.. Physiol Biochem Zool.

[52] Somero GN (2005). Linking biogeography to physiology: Evolutionary and acclimatory adjustments of thermal limits.. Front Zool.

[53] Deutsch CA, Tewksbury JJ, Huey RB, Sheldon KS, Ghalambor CK (2008). Impacts of climate warming on terrestrial ectotherms across latitude.. Proc Natl Acad Sci USA.

[54] Somero GN (2010). The physiology of climate change: how potentials for acclimatization and genetic adaptation will determine the ‘winners’ and ‘losers’.. J Exp Biol.

[55] Sorte CJB, Jones SJ, Miller LP Geographic variation in temperature tolerance as an indicator of potential population responses to climate change.. J Exp Mar Biol Ecol.

[56] Sorte CJB, Hofmann GE (2004). Changes in latitude, changes in aptitudes: *Nucella canaliculata* (Mollusca: Gastropoda) is more stressed at its range edge.. Mar Ecol Progr Ser.

[57] Fields PA, Rudomin EL, Somero GN (2006). Temperature adaptation of cytosolic malate dehydrogenases from native and invasive species of marine mussels (genus *Mytilus*): implications for biogeographic patterning and invasive success.. J Exp Biol.

[58] Braby CE, Somero GN (2006). Following the heart: temperature and salinity effects on heart rate in native and invasive species of blue mussels (genus *Mytilus*).. J Exp Biol.

[59] Grosholz E (2001). Small spatial-scale differentiation among populations of an introduced colonial invertebrate.. Oecologia.

[60] Yund PO, Stires A (2002). Spatial variation in population dynamics in a colonial ascidian (*Botryllus schlosseri*).. Mar Biol.

[61] Grosberg RK (1988). Life-history variation within a population of the colonial ascidian *Botryllus schlosseri*. I. The genetic and environmental control of seasonal variation.. Evolution.

[62] Meehl GA, Tebaldi C (2004). More intense, more frequent, and longer lasting heat waves in the 21st century.. Science.

[63] Sorte CJB, Fuller A, Bracken MES (2010). Increase in non-native species dominance triggered by a simulated heat wave.. Oikos.

[64] Sokolov AP, Stone PH, Forest CE, Prinn R, Sarofirm MC (2009). Probabilistic forecast for twenty-first-century climate based on uncertainties in emissions (without policy) and climate parameters.. J Clim.

[65] Morris RH, Abbott DP, Haderlie EC (1980). Intertidal invertebrates of California..

[66] Lambert CC, Lambert G (1998). Non-indigenous ascidians in southern California harbors and marinas.. Mar Biol.

[67] Cohen AN (2005). Guide to the exotic species of San Francisco Bay.. http://www.exoticsguide.org.

[68] Abbott DP, Lambert CC, Lambert G, Newberry AT, Carlton JT (2007). Ascidiacea. The Light and Smith manual: intertidal invertebrates from central California to Orgeon.

[69] Harris LG, Dijkstra JA (2007). Seasonal appearance and monitoring of invasive species in the Great Bay estuarine system..

[70] Locke A (2009). A screening procedure for potential tunicate invaders of Atlantic Canada.. Aquat Invas.

[71] Ocean Biogeographic Information System.. http://www.iobis.org.

[72] Richardson M (2009). A new invader. Newsletter, Padilla Bay National Estuarine Research Reserve,. http://www.padillabay.gov/newsletter/Winter09-10.pdf.

[73] Locarnini RA, Mishinov AV, Antonov JI, Boyer TP, Garcia HE, Levitus S (2010). World Ocean Atlas 2009, Volume 1: Temperature.. http://www.nodc.noaa.gov/OC5/SELECT/woaselect/woaselect.html.

[74] NOAA (National Oceanic and Atmospheric Administration) (2010). National Oceanographic Data Center Coastal Water Temperature Guide.. http://www.nodc.noaa.gov/dsdt/cwtg/index.html.

[75] Canadian Department of Fisheries and Oceans (2010). Lighthouse Data.. http://www.pac.dfo-mpo.gc.ca/science/oceans/data-donnees/lighthouses-phares/index-eng.htm.

[76] Smithsonian Tropical Research Institute (2010). Environmental Science Program Taboguilla Island Data.. http://striweb.si.edu/esp/index.php.

