# Supplementary figures and images for: Temperature Tolerance and Stress Proteins as Mechanisms of Invasive Species Success
Source: PLoS One. 2011 Apr 26;6(4):e14806. doi: 10.1371/journal.pone.0014806 (PMC3082523; doi:10.1371/journal.pone.0014806)

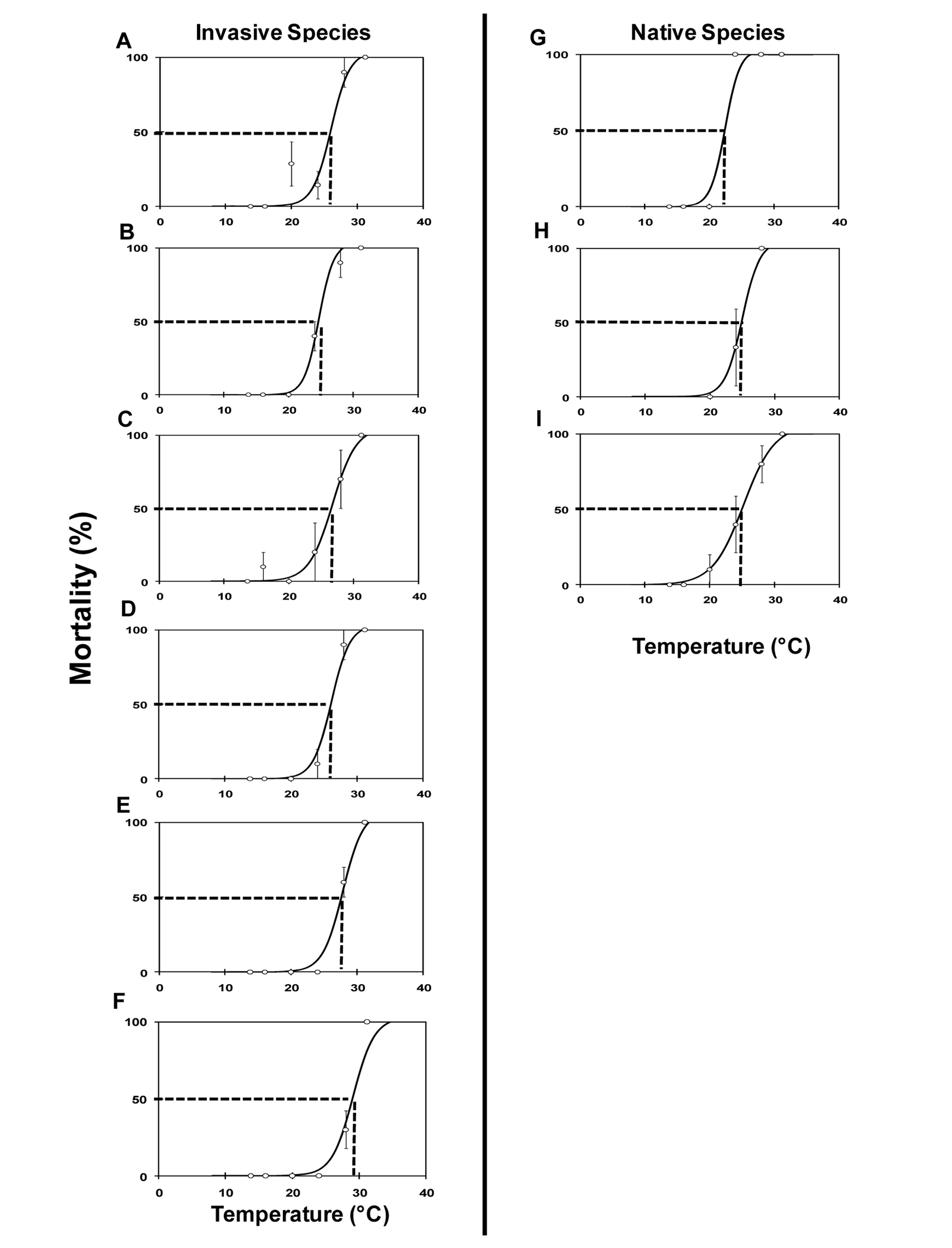

Supplement: Figure S1 — Mean (±1 SE) mortality at 6 treatment temperatures between 14 and 31 degrees Celsius for each species (A, B. neritina; B, Watersipora; C, Botrylloides; D, Didemnum; E, Diplosoma; F, Botryllus; G, Distaplia; H, Ascidia; I, B. californica). LT50 after a 24 h exposure is denoted by the dashed lines. (0.38 MB TIF) [file pone.0014806.s001.tif]
